# Neurological Sequelae in Patients with COVID-19: A Histopathological Perspective

**DOI:** 10.3390/ijerph18041415

**Published:** 2021-02-03

**Authors:** Francesco Fisicaro, Mario Di Napoli, Aldo Liberto, Martina Fanella, Flavio Di Stasio, Manuela Pennisi, Rita Bella, Giuseppe Lanza, Gelsomina Mansueto

**Affiliations:** 1 Department of Biomedical and Biotechnological Sciences, University of Catania, Via Santa Sofia 97, 95123 Catania, Italy; drfrancescofisicaro@gmail.com (F.F.); aldoliberto@gmail.com (A.L.); manuela.pennisi@unict.it (M.P.); 2 Department of Neurology and Stroke Unit, San Camillo de’ Lellis General Hospital, Viale Kennedy 1, 02100 Rieti, Italy; mariodinapoli@katamail.com (M.D.N.); fanella.martina@gmail.com (M.F.); 3 Department of Neurology and Stroke Unit Cesena-Forlì, Bufalini Hospital, AUSL Romagna, Viale Ghirotti 286, 47521 Cesena, Italy; flavds@libero.it; 4 Department of Medical and Surgical Sciences and Advanced Technologies, University of Catania, Via Santa Sofia 87, 95123 Catania, Italy; rbella@unict.it; 5 Department of Surgery and Medical-Surgery Specialties, University of Catania, Via Santa Sofia 78, 95123 Catania, Italy; 6 Department of Neurology IC, Oasi Research Institute-IRCCS, Via Conte Ruggero 78, 94018 Troina, Italy; 7 Department of Advanced Medical and Surgical Sciences (DAMSS), University of Campania “Luigi Vanvitelli”, Piazza L. Miraglia 2, 80138 Naples, Italy; gelsomina.mansueto@unicampania.it

**Keywords:** SARS-CoV-2, COVID-19, neuroinvasion, neuropathology, pathogenesis, autopsy, histopathology, long-term prognosis, outcome

## Abstract

Background: Neuroinvasive properties of SARS-CoV-2 have allowed the hypothesis of several pathogenic mechanisms related to acute and chronic neurological sequelae. However, neuropathological correlates have been poorly systematically investigated, being retrieved from reports of single case or limited case series still. Methods: A PubMed search was carried out to review all publications on autopsy in subjects with “COronaVIrus Disease-19” (COVID-19). Among them, we focused on histological findings of the brain, which were compared with those from the authors’ autoptic studies performed in some COVID-19 patients. Results: Only seven studies reported histological evidence of brain pathology in patients deceased for COVID-19, including three with reverse transcription–quantitative polymerase chain reaction evidence of viral infection. All these studies, in line with our experience, showed vascular-related and infection-related secondary inflammatory tissue damage due to an abnormal immune response. It is still unclear, however, whether these findings are the effect of a direct viral pathology or rather reflect a non-specific consequence of cardiovascular and pulmonary disease on the brain. Conclusions: Notwithstanding the limited evidence available and the heterogeneity of the studies, we provide a preliminary description of the relationship between SARS-CoV-2 and brain sequelae. Systematic autoptic investigations are needed for accurate detection and adequate management of these patients.

## 1. Introduction

The recently emerged Severe Acute Respiratory Syndrome-Coronavirus-2 (SARS-CoV-2) has shown several neuroinvasive properties that have allowed the hypothesis of different pathogenic mechanisms related to both acute and chronic neurological sequelae of the “COronaVIrus Disease-19” (COVID-19). However, the neuropathological correlates have been poorly systematically investigated yet, being essentially retrieved from reports of single case or limited case series still. Moreover, a comprehensive assessment of the neuropathological features observed in the brain of patients deceased for central nervous system (CNS) involvement of COVID-19 is currently lacking.

Here, we reviewed and discussed the current findings from neuropathological examinations performed in deceased patients for COVID-19, with the aim of providing hints for accurate detection and adequate management. We also compared the literature results with the authors’ autoptic studies carried out in some infected patients.

### 1.1. Current Neuropathogenic Hypotheses

In COVID-19, neuroinvasion can take place both in the early and advanced stages [1]. Olfactory mucosa and olfactory bulb can be affected by SARS-CoV-2 entry via the nose, i.e., the transcribrial route. The nasal load can become a source that affects the olfactory bulb and cleft by an initial infection and inflammation around the cells within the olfactory mucosa, causing anosmia. Then, the viral load resulting from rupture of the cells extending from the nasal mucosa to the olfactory bulb via the cribriform plate can be transported by the cerebrospinal fluid (CSF) to the adjacent and distant areas of the CNS. As CSF is present in the subarachnoid space of the meninges directly supporting the olfactory bulb, the virus can reach the CNS without breaching the blood–brain barrier (BBB) [2].

However, earlier pathological and clinical studies have produced conflicting results regarding the presence of the virus in the brain or the CSF. Some authors, indeed, identified the SARS-CoV-2 RNA in autoptic brain exams or the CSF of encephalopathic patients, although at a very low level [3]. Conversely, other studies did not detect any viral sign, although evidence of CSF inflammation was present [4,5], suggesting an artifact or contamination [6].

At the structural level, surface glycoproteins of Coronaviruses (CoVs), such as the spike protein, are composed of 14 binding residues directly interacting with the human angiotensin-converting enzyme-2 (ACE2) receptor. ACE2 is also used as the main docking receptor by SARS-CoV-2, which needs a proteolytic process of the spike protein by the transmembrane protease serine-2 for an effective cell attack [7]. Therefore, ACE2 is not only an enzyme but also a functional surface receptor through which SARS-CoV-2 enters the host cells and highly expressed in the heart, kidneys, lungs, and blood cells. ACE2 is also a key regulator of the renin–angiotensin–aldosterone system (RAAS). SARS-CoV-2 can cause an ACE/ACE2 balance disruption and RAAS activation, which eventually leads to disease progression, especially in patients with comorbidities, such as hypertension, diabetes mellitus, and cardiovascular diseases [8].

The BBB may represent the second modality of entry of SARS-CoV-2, and two main theories have been hypothesized for the BBB crossing. The first mechanism would act through a cellular transport across vascular endothelium [9]; as soon as the virus attacks vascular and neuronal cells, it would interact with the ACE2 on the vessels, neurons, and glia, then starting a budding cycle that further damages both neuronal and vascular tissues [1]. The second hypothesis relies on the so-called “Trojan horse mechanism”, through the infection of leukocytes passing across the BBB [10]. Since the ACE2 is expressed by granulocytes, monocytes, and lymphocytes, SARS-CoV can infect them [11,12,13,14], and SARS-CoV-2 might act with the same modality. Furthermore, systemic inflammation related to COVID-19 would enhance the BBB permeability, thus promoting CNS invasion by the immune cells infected [15].

Lastly, it cannot be excluded that SARS-CoV-2 may persist in some CNS resident cells and clinically act as a cofactor of clinical exacerbations. Indeed, CoVs have been found in neurological disorders, such as multiple sclerosis (MS), optic neuritis, and Parkinson’s disease [16,17,18,19]. In parkinsonian patients, CSF was blinded analyzed by the enzyme-linked immunosorbent assay for antibody response to four CoV antigens (mouse hepatitis virus JHM and A59, and human coronavirus 229E and OC43) [16], whereas in MS patients, intrathecal antibody synthesis to OC43 and 229E viruses was detected in 41% and 26%, respectively [18]. Accordingly, it was suggested that the persistence of CoV infection might pathogenically contribute to the onset and course of these disorders. For instance, it is known that some infective agents may trigger MS, viruses being the most likely involved in some genetically predisposed patients [20]. Conversely, among CSF from 37 subjects with optic neuritis, only four patients and one control were positive for CoV 229E and none for OC43, thus not providing evidence for an etiological role of human CoVs [19].

**Figure 1:**
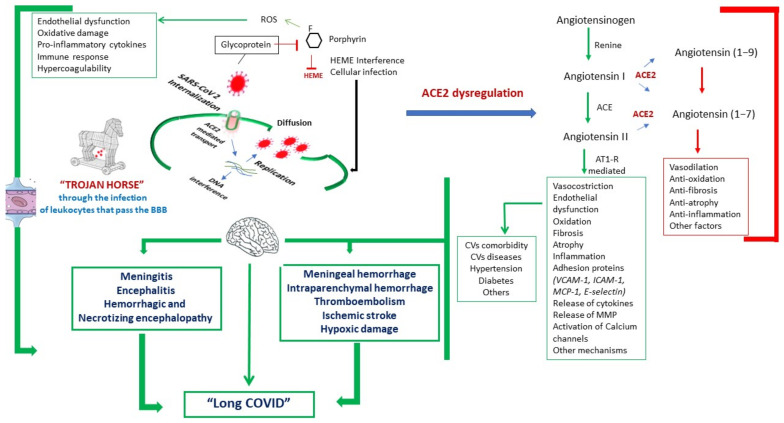
Main pathophysiological mechanisms proposed in patients with COronaVIrus Disease-19 (COVID-19) and related acute and long-term neurological manifestations. Angiotensin-converting enzyme-2 (ACE2) and iron chelated with any porphyrin, irrespective of the valence state of the iron atom (HEME) dysregulation, as well as direct action on vascular endothelium, immune response, and inflammation (with the subsequent release of inflammatory mediators), and the “Trojan horse hypothesis” (i.e., leukocytes-carriers of infection through the blood–brain barrier) have been proposed as the main mechanisms responsible for brain lesions. The inhibitory pathways are shown in red, while the activatory pathways are indicated in green, along with morphological changes responsible for cardiovascular and brain diseases, especially in comorbid patients. *Legend:* ACE2: angiotensin converting enzyme-2; AT1-R: angiotensin II receptor type; BBB: blood–brain barrier; CVs: cardiovascular manifestations; HEME: iron chelated with any porphyrin, irrespective of the valence state of the iron atom; ICAM-1: intercellular adhesion molecule 1; MCP-1: monocyte chemoattractant protein-1; MMP: matrix metalloproteinase; ROS: reactive oxygen species; VCAM-1: vascular cell adhesion molecule 1.

Figure 1 summarizes the main pathophysiological mechanisms proposed in patients with COVID-19 and acute and long-term CNS involvement.

### 1.2. Overview of the Main Neurological Manifestations

Although neurological complications may arise from a direct effect of SARS-CoV-2, they usually reflect a systemic response to the infection, with severe cases of COVID-19 more often producing CNS complications with respect to mild forms (45.5% vs. 30%) [21]. Moreover, most of these patients are in the older age group, and exhibit comorbidities, especially hypertension, and the neurological involvement can occur independently of the respiratory manifestations [22,23].

Globally, asthenia, myalgia, headache, anosmia, and ageusia are the most common symptoms, followed by encephalopathy, stroke, and seizures [9]. In extreme cases, some patients may exhibit encephalitis, flaccid paraparesis, and coma [22]. Of note, telemedicine is no longer a futuristic concept, being the new normal for an increasing number of medical and surgical specialists during the pandemic [24].

A recent meta-analysis concluded that among 70 patients with COVID-19 and neurological manifestations (mean age 61.9 ± 17.7 years, 60.6% male), 39 (53.4%) had a stroke, 18 (24.7%) a Guillain–Barré syndrome or its variants, 11 (15.1%) encephalopathy, meningitis, encephalitis, or myelitis, and 5 (6.8%) seizures. Neurological disorders presented after 8.1 ± 6.8 days from the onset of infection. The average rate of mortality was 17.8% (mortality rate of stroke 25.6%), whereas chemosensory dysfunction occurred in 59.9% and 57.5% of patients with anosmia and ageusia, respectively [25].

More recently, a systematic search on 19 studies and >12,000 adult patients with laboratory-confirmed COVID-19 [26] found that headache was reported in 7.5% of patients, dizziness in 6.1%, hypo-anosmia and gustatory dysfunction in 46.8% and 52.3%, respectively, whereas symptoms of muscular injury ranged between 15% and 30%. Three studies reported a radiologically-confirmed acute cerebrovascular disease in 2% of patients [26].

Older subjects or those with pre-existent cognitive impairment, several comorbidities, past medical conditions, poor pre-morbid functional independence, malnutrition, concomitant infections, or multiple vascular risk factors are also at higher risk for altered consciousness and encephalopathy when affected by COVID-19 [27,28,29]. Endocrine or metabolic disorders, such as hypo-hypercalcemia, hypo-hypernatremia, hypo-hyperglycemia, liver and/or kidney dysfunction, and sepsis confer a further risk [30].

An acute hemorrhagic necrotizing encephalopathy (AHNE) was described in a COVID-19 patient [31]. Brain magnetic resonance imaging revealed multifocal and symmetric contrast-enhanced hemorrhagic lesions in the thalamus, mesial temporal region, and insula, bilaterally [31]. A similar case occurred in a previously healthy 44-year-old woman [32]. A posterior reversible encephalopathy-like syndrome, with reversible cortical blindness [33] and mild encephalopathy, was also described [34]

Older individuals with COVID-19 are at higher risk for neurovascular events [35]. In a retrospective cohort of 221 subjects [36], eleven (5%) underwent ischemic stroke, one (0.5%) cerebral hemorrhage, and one (0.5%) cerebral venous thrombosis. Other authors reported five patients with stroke (of ischemic origin in four of them) in the context of severe COVID-19, thrombocytopenia, increased D-dimer, and multiple organ failure [29]. Of note, a previous investigation in the USA observed that young persons (<50 years) more likely underwent large-vessel strokes during the course of COVID-19, thus showing that all age groups can be affected [37]. Increased markers of inflammation and a state of hypercoagulability seem to be a feature of severe forms [29]. Indeed, systemic inflammation associated with the infection, vasculitis, and thrombosis is known to enhance the risk of stroke [38]. Finally, both systemic vasculitis and CNS vasculitis were autoptically documented in SARS-CoV patients [39].

Lastly, smell and taste disorders are often complained about by COVID-19 patients and can manifest suddenly [40]. In Italy, 19.4% of subjects had some forms of anosmia and ageusia [41], and in a registered study on 12 European centers, including 417 patients with mild-to-moderate COVID-19, chemosensory dysfunction had a prevalence of 85.6% (anosmia) and 88% (ageusia) [42]. Notably, olfactory dysfunction was mentioned as the onset symptom in 12% of them, without any runny nose or nasal obstruction in 18% [42].

### 1.3. Autopsy, Brain Sampling, and Histopathological Assessment in COVID-19 Patients

Unlike other organs and tissues, which can be easily sampled by biopsy, a comprehensive histopathological assessment of the nervous system requires a detailed examination of both the CNS and peripheral nervous system (PNS). At the onset of the pandemic, several countries, including Italy, did not allow autopsies in the attempt to contain the risk of infection and its spread. Later, although many local governments and scientific communities appreciated the missed opportunity of collecting histopathological data, the number of autopsies has only increased slowly [43], probably due to the limited pathologists trained in the brain and the few centers able to guarantee the safety of the mortuary staff. According to the international guidelines [44,45,46], indeed, autopsies must be performed within a certified biosafety level 3 Autopsy Room, and mortuary staff must be equipped with personal protection equipment (disposable headgear, double pair of disposable gloves, cut-resistant protective gloves, respiratory filter FFP3 protection, Tyvek coverall suits). During brain and spinal cord removal, particular care is also needed to avoid bone aerosolization.

Another relevant concern regards the sampling. It is often unclear if it has been sufficient and if standard protocols have been adopted; therefore, the microscopic results obtained may not be comparable [47]. Furthermore, data appear to be heterogeneous since only a few papers have identified the virus with the reverse transcription–quantitative polymerase chain reaction (RT-qPCR). Even when the assay was executed, it is also uncertain whether the genome could be exclusively identified with that of SARS-CoV-2 [47].

To date, the management of neurological symptoms and therapy of COVID-19 patients seem to be more challenging than pulmonary and cardiovascular manifestations, those being more systematically investigated [48,49,50,51,52,53]. Furthermore, in the pandemic scenario, some symptoms, and signs of CNS involvement, e.g., those underlying meningitis or encephalitis, were not always correctly diagnosed and promptly managed. Therefore, the questions we want to draw attention to are the following: How many cases were actually attributed to a direct invasion of the CNS by SARS-CoV-2? How many cases have been not diagnosed or misdiagnosed due to a failure to identify the virus in the CSF or at the post-mortem examination? Can we make a reliable differential diagnosis between direct viral damage and superposition damage by other causes or etiological agents?

To properly answer these questions, it should be preliminarily stated that, although autopsies are still scarce compared to the entity of the pandemic, the most common pathological lesions in COVID-19 appear to be similar to those seen in the context of other viral and systemic diseases associated with cardiovascular involvement. Based on our autopsy experience [54,55,56,57,58] and current evidence from the literature, it seems that morphological data are still limited to a few cases of meningitis or necrotizing encephalopathy [3,30,58]. More recently, it has also been hypothesized that neuropathological changes might be the consequence of direct or immune-mediated damage of the virus, although a secondary lesion from the interaction through the ACE2 receptor or interference with the HEME formation cannot be excluded (Figure 1), as well as an indirect injury from a systemic dysfunction [50,59,60,61,62,63]. Unbalanced homeostasis, immune-mediated action, and damage from cytokines release and inflammatory mediators (i.e., the “cytokine storm”) seem to contribute to neurological deficits [50,59,60,61,62,63]. It is also likely that hypoxemia plays a role in many patients with encephalopathy, it being secondary to metabolic derangements due to multiple organ failure or medication effects.

Nevertheless, it should be taken into account that peripheral biomarkers showed non-specific signs of neuronal damage and reactive gliosis in moderate-to-severe patients with COVID-19. This does not seem to suggest a distinctive neuropathogenesis of SARS-CoV-2 [64]. Similarly, weak evidence supports direct viral damage, which presupposes its capacity for overcoming the BBB. Moreover, SARS-CoV-2 can infect different cell types, and data on the involvement of endothelial cells alone are enough to explain most of the pulmonary, cardiovascular, and other organ damage, including the CNS. Additional data come from the few pathological findings described in subjects with SARS-CoV and Middle East Respiratory Syndrome (MERS)-CoV: histologically, edema and non-specific neuronal degeneration were mostly observed at patients’ autopsies [11,39,65,66,67].

## 2. Data Sources and Selection

A PubMed (MEDLINE) review was carried out to identify all studies dealing with autopsies performed in COVID-19 patients. The following search terms were used, from database inception to December 2020: COVID-19, SARS-CoV-2, autopsy, neuropathology, histology, morphology, encephalopathy, ischemic stroke, intracerebral hemorrhage, gliosis, necrotizing encephalopathy, encephalitis, myelitis, and inflammation.

A total of more than 11,000 articles, including those listed in the references of the retrieved studies, were found originally. We then excluded the following items: all publications not dealing with COVID-19 autopsy or neuropathology; all studies different from original articles (e.g., reviews, case report/case series, letters, commentaries, etc.); all preclinical studies or research performed on animals or cell cultures; non-English written papers; any other publication that did not comply with the goal of the present review. After this process, 46 studies performed autopsy: 39 showing pulmonary, cardiovascular, or other organ involvement (Appendix A), and 7 showing brain pathology (Table 1). Among them, only 3 autopsy studies demonstrated a RT-qPCR-positive viral infection [68,69,70].

## 3. Results and Discussion

### 3.1. Main Findings

**Figure 2:**
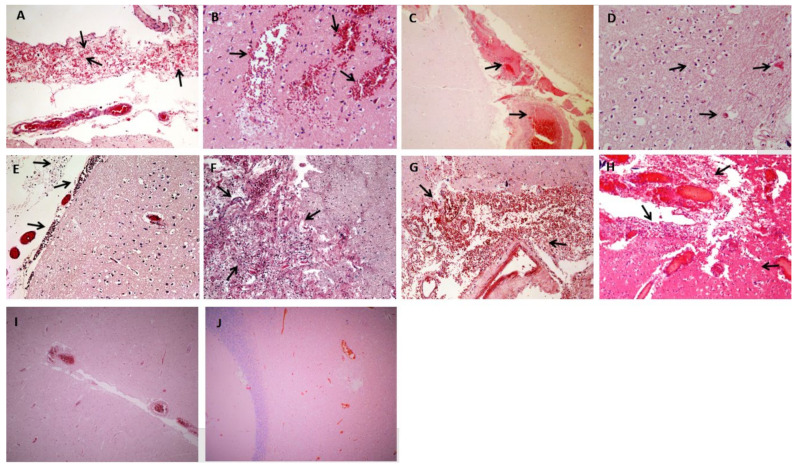
Main anatomopathological findings from authors’ autoptic studies carried out in some COVID-19 patients. (**A**–**D**): vascular-related changes; (**A**): meningeal erythrocyte extravasations (arrows) [Hematoxylin and eosin (H&E) stain ×10]; (**B**): focal and multiple intraparenchymal erythrocyte extravasations (arrows) (H&E stain ×40); (**C**): fibrinoid material aggregated in the vessel lumen (thrombo-embolus), with associated pale parenchymal areas as from ischemic damage in the first phase (arrows) (H&E stain ×10); (**D**): initial transformation of neuronal cytoplasm and nucleus with the presence of pink neurons from hypoxia (arrows) (H&E stain ×40). (**E**–**H**): infection-related changes (H&E stain ×20); (**E**): inflammatory lymphomonocyte infiltrate in meningitis (arrows); (**F**): area of inflammatory infiltrate with neuronal destruction and reactive aspects (arrows); (**G**,**H**): combined aspects that are often associated in hemorrhagic (**G**) and necrotizing (**H**) encephalopathy, characterized by erythrocyte extravasations, mainly in perivascular regions, with areas of adjacent intraparenchymal necrosis and more or less evident inflammatory rate (arrows). (**I**,**J**): normal brain findings (H&E stain ×10); (**I**): cerebral cortex, near a sulcus; (**J**): cerebral cortex, near the hippocampus.

Table 1 and Appendix A report the main autopsy studies during the COVID-19 pandemic. As expected, pulmonary and cardiovascular lesions were those investigated the most and basically appeared not to be different from those previously described in SARS-CoV and MERS-CoV [51,52,53]. Diffuse alveolar damage, myocarditis, myocardial infarction, thromboembolism, disseminated intravascular coagulation, among others, have been reported. Of note, thromboembolism and ischemic stroke have been explained in the context of acute events affecting the cardiovascular system. Large vessel occlusion by hypercoagulability and cerebral vasculitis were also common [74]. Taken together, these findings are in line with the authors’ experience (Figure 2).

It clearly emerges from these studies that the CNS represents a relevant target in the disease course of some patients [47], whose brain might be affected by both vascular-related changes and/or infection-related changes. Indeed, the consensus seems to converge on a little or no direct COVID-19 brain damage (i.e., “pure” encephalitis), a relevant contribution of thrombo-embolic vascular changes, a still debatable consequence of the “cytokine storm” in very ill elderly persons, and a few but severe encephalopathic/encephalitis syndromes of unknown causation, although rarely found to be common or representative. Nevertheless, only few of the radiologically and histopathologically identified lesions can be clearly attributed to a vascular injury, usually in the context of vascular comorbidities or risk factors predisposing to ischemic or hemorrhagic conditions. Therefore, performing a brain autopsy in COVID-19 is relevant for the differential diagnosis between a secondary vascular injury related to SARS-CoV-2 infection and primary direct virus damage [47].

Up to now, the few autopsy studies focused on the neurological involvement of COVID-19 may be ascribed to the relatively few deaths from neurological complications compared to those from pulmonary or cardiovascular disease, as well as to purely technical-procedural reasons or safety-related issues to biopsy or extensive organ examination. Therefore, these considerations should be taken into account when considering the global epidemiology and the estimation of neurological complications causing death in COVID-19 and the effort to make a clear distinction between direct damage to the CNS from a secondary cerebral lesion due to associated comorbidities and systemic viral dissemination. Moreover, recent clinical reviews demonstrated that SARS-CoV-2 affects not only the CNS but also the PNS and muscles [66,75,76,77,78].

Based on the clinical studies and the limited neuropathological data, CNS histopathology encompasses hypoxic/ischemic encephalopathy, activated microglial cells with variable infiltration of perivascular T lymphocyte, cerebrovascular diseases, AHNE, encephalitis/meningitis, acute myelitis, demyelinating disorders, and PNS involvement [21,68,71,73,79,80,81,82,83,84,85,86,87,88,89,90,91,92,93,94,95,96,97,98,99,100,101,102,103,104,105,106,107,108,109,110,111,112,113,114,115], with histologically documented lesions in some studies [6,11,68,69,71,72,73].

Hypoxic/ischemic encephalopathy causes several changes to the brain secondary to a combination of neuroinflammatory response, decreased cerebral oxygen supply due to pneumonia during the acute phase of infection, and systemic metabolic disorders. Accordingly, CoV infections, including that by SARS-CoV-2, have been diffusely described in association with a “cytokine storm syndrome”. Clinically, symptoms are dysphoria, headache, delirium, confusion, mental disorder, loss of consciousness, and coma in severe cases. This histopathological pattern seems to be the most common correlate in COVID-19 patients with clinical CNS involvement [116].

Regarding acute neurovascular accidents and their sequelae, SARS-CoV-2 is able to induce a systemic inflammatory response and a hypercoagulable state, as indexed by an increase in D-dimer, a prolongation of the prothrombin time, and disseminated intravascular coagulation, which confer a greater risk of ischemic and/or hemorrhagic complications [117]. Severe COVID-19 cases, therefore, are at high risk of thrombosis secondary to an indirect hypercoagulable state and a direct vascular endothelial damage [116]. In AHNE, coagulative neuronal necrosis, reactive gliosis, and perivascular lymphocytic infiltration with cell debris or inclusions were the main histological aspects [68].

Regarding meningitis and encephalitis, albeit viral encephalitis was expected to be one of the most common complications, the current evidence indicates that it is quite rare in COVID-19. In the encephalomyelitis/acute disseminated perivascular encephalomyelitis, processes of acute inflammation involving the CNS and causing demyelination were described in association with neuronal damage and some vascular lesions, such as multifocal hemorrhage and/or micro-infarcts and perivascular inflammation with histiocytes and astrocytosis infiltration. Up to now, however, clinically diagnosed cases are very scarce, although those reported were not confirmed at the histopathological exam. In general, it is also difficult to diagnose viral CNS diseases, mainly because of symptom variability, a paucity of specific markers, and challenges in distinguishing it from other viral or even non-viral encephalitis, as well as from a non-specific encephalopathy related to a systemic viral infection [116].

Since demyelination is usually caused by the autoimmune reaction triggered by an excessive and widespread inflammation [72], there are also findings of demyelinating lesions induced by SARS-CoV and associated with MS earlier in the pandemic. However, one case only histologically documented neuronal damage with perivascular lymphocytes and microglial reaction, associated with the CD68+ histiocytic population more represented than lymphocytes [71]. Moreover, pathological correlates are not strong, thus requiring more and systematic investigations in this “cutting-edge” topic. Finally, reduced T lymphocytes and lymphopenia in COVID-19 have been described, a finding which may put vulnerable individuals at risk for opportunistic infections, especially if this occurs in combination with steroid treatment. However, additional evidence is needed to support the vulnerability of subjects with COVID-19 to opportunistic infections [116].

### 3.2. Comments and Outlooks

Overall, neuropathological changes in patients with COVID-19 appear to be of a mild entity, neuroinflammatory signs being the most common finding, along with reactive gliosis, astrocytosis, and microglia activation [70].

While COVID-19 patients continue to be reported, the neuropathological pattern of ischemic/hypoxic injury, cerebral hemorrhage, and mild or moderate non-specific inflammation are unlikely to vary [118] significantly. The identification of CoVs particles by electron microscopy is also difficult because of the similar appearance of normal cell structures, an aspect which has generated several controversies [118]. The inherent bias of autoptic exams for severe, fatal cases and institutional restrictions for morphological CNS assessment have also determined that some studies might have overestimated both the extent and frequency of neuropathological findings, whereas others might have underestimated them [119]. Finally, autopsies in children, which include the multisystem inflammatory syndrome, remain extremely rare. Although they are responsible for <2% of all COVID-19 [120], neuropathological data from pediatric patients can significantly aid in disentangling the peculiar pathophysiological basis underlying the pediatric disease, including the typical immune-response, the degree of ischemic/hypoxic damage, and the hypercoagulability state of this age-group.

Further “hot topics” are the characterization of the effects of antiviral therapies (such as remdesivir), steroids (including dexamethasone), immunomodulatory medications (e.g., anti-interleukin-6), monoclonal antibodies, and anticoagulant agents on the brain [119]. Since therapeutic responses to COVID-19 vastly differ among centers, the understanding of how the different treatment options during hospitalization and after discharge might be responsible for the variability in both acute and long-term neurological manifestations and their histopathological correlates [121] remains. Regarding long-term sequelae, neuropathological findings in COVID-19 survivors are totally unstudied. Some CNS symptoms, such as asthenia, headache, myalgia, anosmia, and dysgeusia, may last for weeks or even months in a non-negligible portion of patients [122,123], and studies determining causes and mechanisms underlying long-term manifestations are required.

The persistence of symptoms or development of new symptoms related to SARS-CoV-2 infection late in the course of COVID-19 is an increasingly recognized problem facing the infected population and health systems. “Long-COVID” or “COVID long-haulers” describes those persons who experience symptoms for more than 28 days after the diagnosis of SARS-CoV-2 infection [124]. Symptoms are as markedly heterogeneous as seen in acute disease and may be constant, fluctuating, or replaced by other symptoms of varying frequency and severity. Such multisystem involvement requires a holistic approach and, although many patients can be managed in primary care, others need rehabilitative care [124]. In particular, a significant proportion of patients report persistent and debilitating symptoms centered not only around fatigue but also including “brain fog”, pain, breathlessness, and dysrhythmias, that extend several months in the post-infection period [125]. These symptoms are a characteristic of a well-documented and largely unexplained post-viral illness, i.e., the myalgic encephalomyelitis/chronic fatigue syndrome (ME/CFS).

The “long haulers” with ME/CFS, who do not make a straight-forward recovery in the post-viral period, probably reflect damage by the host response to the initial infection [126]. A severe host response (e.g., the “cytokine storm”), indeed, can give rise to oxidative and inflammatory damage and generalized oxidative stress. Pathophysiological changes in SARS-CoV-2 that enhance the production of reactive oxygen species might be ameliorated by free radical scavengers, also confirming that oxidative stress and SARS-CoV-2 pathogenesis may be closely linked [127] and that some antioxidant therapies might be of beneficial effect [128].

### 3.3. Limitations

The few studies available, which also differ in terms of methodology, is the main limitation of this review. Thus, the generalization of the findings reviewed here requires further validation given the low number of cases, the limited clinical data, and the lack of sex- and age-matched controls. To date, therefore, the etiological link between SARS-CoV-2 infection and autoptic brain correlates remains inconclusive. In particular, the possibility of primary viral-related damage on neurons and glia needs to be validated by systematic identifications of the viral genome within the brain or at the CSF. Surely, the triggering of an abnormal immune response, together with the release of neurochemical factors and inflammatory mediators, significantly contribute to BBB disruption, changes in brain homeostasis, and clinical manifestations.

Other critical aspects regard the sensitivity and specificity of the diagnostic techniques used, the unavoidable effect of unspecific post-mortem phenomena on brain tissue, and the challenges in studying a large number of people or particular age groups (such as children and young adults).

Further, larger and independent works on autoptic exams, integrated with clinical, laboratory, CSF, imaging, and general autoptic data, are needed to elucidate the impact of SARS-CoV-2 on CNS and its role in the process, progression, and mortality of COVID-19.

## 4. Conclusions

Notwithstanding the limited evidence available and the heterogeneity of the studies, in this review, we provided a preliminary description of the complex relationship between SARS-CoV-2 and CNS sequelae. Despite the exponentially increasing number of publications on COVID-19, the lack of well-defined pathophysiology and systematic neuropathology of long-term neurological consequences of SARS-CoV-2 still stands. Given the peculiar features of the CNS and the challenges in collecting samples, autopsy remains the gold standard exam for studying any primary CNS disorder and its involvement in the course of systemic diseases, such as COVID-19. More autopsies would mean further knowledge, accurate diagnosis, new therapeutic approaches, and improvement in the survivors’ quality of life. In this tragic pandemic, lessons from dead people can rescue lives.

## Figures and Tables

**Table 1:** Relevant autopsy studies performed during COronaVIrus Disease-19 (COVID-19) pandemic.

Study [References]	*n*; Sex	Age (Years)	P/CV/C	RT-qPCR+	CNS Involvement
Stoyanov et al., 2020 [68]	1; F	77	√	√	Acute necrotizing encephalitis
Al-Dalahmah et al., 2020 [71]	1; M	73	√		Neuronal damage Neuronophagy Perivascular lymphocytes Microglial reaction
Paniz-Mondolfi et al., 2020 [69]	1; M	74		√	Cerebrospinal fluid negative No data about the detection of histological lesion
Reichard et al., 2020 [72]	1; M	71			Acute disseminated perivascular encephalomyelitis Hemorrhagic lesions Microinfarctions
Bryce et al., 2020 [73]	67 (20 brains); 38 M	Median: 69.0 IQR: (34.0–94.0)	√		Disseminated micro thrombosis Ischemic infarcts Hemorrhage
Solomon et al., 2020 [6]	18; 14 M	Median: 62.0 IQR: (53.0–75.0)			Acute cerebral and cerebellar hypoxia-related lesions Neuronal loss within the cerebral cortex, hippocampus, and cerebellar Purkinje cell layer Perivascular lymphocytes Focal leptomeningeal inflammation
Matschke et al., 2020 [70]	43; 27 M	Median: 76.0 IQR: (70.0–86.0)		√	Ischaemic lesions Astrocytosis Microglia activation Cytotoxic T lymphocytes at the brainstem, cerebellum, basal ganglia, and olfactory bulb

Legend: *n*: number of patients; F: female; M: male; CNS: Central Nervous System; IQR: interquartile range; P: pulmonary involvement; CV: cardiovascular involvement; C: coagulopathy; RT-qPCR: reverse transcription–quantitative polymerase chain reaction.

## Appendix A

**Table A1:** Autopsy studies in COVID-19 individuals without brain involvement.

Study	P/CV/C	Other Organs
Tian et al., 2020 [80]	√	
Danzi et al., 2020 [79]	√	
Su et al., 2020 [81]		Kidney
Peleg et al., 2020 [82]		Kidney
Zhang et al., 2020 [83]	√	
Yao et al., 2020 [84]	√	Kidney, spleen, liver
Li et al., 2020 [85]	√	
Rossi et al., 2020 [86]		Kidney
Wichmann et al., 2020 [87]	√	
Shanes et al., 2020 [88]		Placenta
Hosier et al., 2020 [89]		Placenta
Varga et al., 2020 [90]	√	Kidney
Dolhnikoff et al., 2020 [91]	√	
Magro et al., 2020 [92]	√	Skin
Buja et al., 2020 [93]	√	
Ducloyer et al., 2020 [94]	√	
Lax et al., 2020 [95]	√	
Suess and Hausmann 2020 [96]	√	
Flikweert et al., 2020 [97]	√	
Li et al., 2020 [98]	√	
Sonzogni et al., 2020 [99]	√	Liver
Carsana et al., 2020. [100]	√	
Bradley et al., 2020 [101]	√	Spleen
Schaller et al., 2020 [102]	√	
Remmelink et al., 2020 [103]	√	
Nunes Duarte-Neto et al., 2020 [104]	√	
Prilutskiy et al., 2020 [105]	√	
Xu et al., 2020 [21]	√	
Konopka et al., 2020 [106]	√	
Yan et al., 2020 [107]	√	
Fitzek et al., 2020 [108]	√	
Rev Esp Patol. 2020 [78]	√	
Luo et al., 2020 [109]	√	
Tian et al., 2020 [110]	√	
Kuang et al., 2020 [111]	√	
Pernazza et al., 2020 [112]	√	
Fox et al., 2020 [113]	√	
Barton et al., 2020 [114]	√	
Brook et al., 2020 [115]	√	

Legend: P: pulmonary involvement; CV: cardiovascular involvement; C: coagulopathy.
